# A single-nucleotide mutation of G301A in *GaIAA14* confers leaf curling in *Gossypium arboreum*

**DOI:** 10.3389/fpls.2025.1645239

**Published:** 2025-07-22

**Authors:** Pengfei Miao, Huan Zhang, Yifan Xu, Ruowen Zhang, Yunfei Hao, Guoli Song, Ji Liu

**Affiliations:** ^1^ National Nanfan Research Institute (Sanya), Chinese Academy of Agricultural Sciences, Sanya, China; ^2^ State Key Laboratory of Cotton Bio-breeding and Integrated Utilization, Institute of Cotton Research, Chinese Academy of Agricultural Sciences, Anyang, China

**Keywords:** *Gossypium arboreum*, *CU*, *GaIAA14*, G301A, curly leaf

## Abstract

Cotton is a crucial fiber and oil crop, playing a significant role in the textile and food industries. Its yield heavily relies on photosynthesis, a process that primarily occurs in the leaves. Consequently, leaf morphology stands as a vital agronomic trait in cotton breeding. However, research on the molecular mechanisms underlying cotton leaf morphogenesis remains relatively limited. Here we identified a curly leaf mutant (*CU*) in *Gossypium arboreum* by ethyl methylsulfonate (EMS) mutagenesis. The genetic analysis revealed that the curly leaf trait in this mutant is a semi-dominant characteristic controlled by a single gene. The map-based cloning of the *CU* locus showed a single-nucleotide mutation from G to A at the 301st positions in *AUX/IAA14* protein (*GaIAA14*), which resulted in an amino acid substitution from valine (V) to isoleucine (I). After silencing *GaIAA14* through virus-induced gene silencing (VIGS) technology in *CU* mutant, the leaves exhibited a flattened phenotype, indicating that *GaIAA14* is a key gene regulating leaf curling in cotton. Comparative transcriptomic RNA-Seq analysis revealed significant changes in the expression levels of most auxin-related genes, suggesting that the mutation disrupts auxin signaling transduction. These findings establish a foundation for further functional studies of this gene and provide research strategies for leaf morphology improvement.

## Introduction

Leaf shape is one of the most significant agronomic traits, which plays a crucial role in photosynthesis and crop yield (Zang et al., 2024). Proper leaf curling is beneficial to form a more upright plant architecture, which enhances the photosynthetic efficiency of the canopy and reduces water loss through transpiration, thereby improving plant growth and stress resistance (Li et al., 2017).

Auxin plays an important role in leaf morphogenesis. The auxin/indole-3-acetic acid (Aux/IAA) gene family functions as an early auxin response factor and a transcription repressor that regulates the transcription of downstream genes of the auxin pathway. At high auxin concentrations, AUX/IAA proteins form the AUX/IAA-auxin-SCF^TIR1/AFB^ complex, which leads to AUX/IAA proteins’ ubiquitination and degradation (Maraschin et al., 2009). At low auxin concentrations, AUX/IAA proteins form ternary complexes with ARF and TPL that bind to the promoters of auxin-regulated genes and inhibit their transcription (Szemenyei et al., 2008).

The AUX/IAA protein domain II possesses a conserved GWPPV (I) motif, termed the degradator, which binds to TIR1-type SCF and regulates AUX/IAA protein degradation. The amino acid variations in GWPPV (I) usually lead to dominant or semi-dominant gain-of-function mutations (Gray et al., 2001). In *Brassica napus*, mutations of the GWPPV motif to GWSPV or GWPSV in the IAA2 protein (Tan et al., 2023; Huang et al., 2020) and mutations of GWPPV to EWPPV or EWLPV in the IAA7 protein (Wei et al., 2021; Cheng et al., 2019), as well as the mutation of GWPPV to EWPPV in the IAA8 protein of *Arabidopsis* (Wang et al., 2013), all resulted in leaf curling in plants. Overexpression of the *IAA27* gene in both soybean and blueberry resulted in leaf curling (Su et al., 2022; Hou et al., 2020). Ectopic expression of the poplar auxin repressor gene *PtrIAA14.1* in *Arabidopsis* resulted in pleiotropic auxin hypersensitivity phenotypes, including downward leaf curvature, enhanced shoot branching, and severely compromised fertility (Liu et al., 2015). Similarly, the heterologous expression of *M.* sp*icata MsIAA32* in *Arabidopsis*, which lacks the TIR1-binding domain, resulted in auxin-deficient phenotypes, including epinastic leaves and suppressed lateral root development, consistent with its role as a non-degradable AUX/IAA repressor (Reddy et al., 2024). These findings demonstrate that the degron motif “GWPPV(I)” is essential for the stability of AUX/IAA proteins. The increased abundance of AUX/IAA proteins disrupts auxin signaling, resulting in auxin insensitivity and auxin-deficient phenotypes.

Leaf morphogenesis proceeds through three coordinated stages: initiation of leaf primordia, specification of adaxial–abaxial polarity, and marginal meristem activity (Du et al., 2018). Leaf flattening primarily depends on the establishment of adaxial–abaxial polarity—a process governed by a complex regulatory network. Any defect or disruption in adaxial–abaxial polarity establishment will induce leaf curling in plants (Ha et al., 2010; Zhan et al., 2025). In cotton, leaf curling phenotypes are categorized into three types: rolling leaf, curly leaf, and cup leaf (Di et al., 2016). The curly leaf mutants of Asian cotton (*Gossypium arboreum*) ‘Xiaobaihua’ and upland cotton (*Gossypium hirsutum*) ‘Stoneville 2B’ are each controlled by a single recessive gene, exhibiting upward and inward curling of leaf margins (Yu, 1939; Di et al., 2016). Map-based cloning identified *Gh_A11G2653* (an ortholog of *Arabidopsis CCT8*) as the gene underlying cup-shaped leaves in a cotton mutant T582 (Zang et al., 2024). CCT (chaperonin containing T-complex protein) belongs to the molecular chaperone family and regulates protein folding and assembly in cells (Blanco-Touriñán et al., 2021). The *Arabidopsis cct8* mutant also exhibits cup-shaped leaves (Xu et al., 2011), indicating functional conservation between cotton *Gh_A11G2653* and *Arabidopsis CCT8*. Further studies revealed that mutations in *CCT8* (designated *GHCU*) disrupt the transport of the HD protein KNOTTED1-like (*KNGH1*) from the adaxial to abaxial domain, leading to asymmetric auxin distribution and consequent upward-curling leaf phenotypes (Zang et al., 2024). However, the mechanism of AUX/IAA proteins in cotton leaf curling is still largely unknown. Here we mutated the *G. arboreum* cultivar Shixiya 1 with EMS and screened a curly leaf mutant**—** *CU*. Genetic analysis and gene mapping confirmed that the *CU* mutant phenotype is a semi-dominant inherited trait controlled by *GaIAA14.* These findings accelerate the deeper functional characterization of *IAA14* and offer mechanistic insights for the improvement of cotton plant architecture.

## Materials and methods

### Plant materials and growth condition

*Gossypium arboreum* line Shixiya 1 originated in South China and was subsequently introduced to the Yangtze and Yellow River regions. Shixiya 1 seeds were used for EMS treatment. The *CU* mutants were obtained from EMS mutagenesis, which possess a stable inheritable leaf curling trait. *Gossypium arboreum* lines DQJ and BML were used as male parent lines to construct the F_2_ population. All of the plants were sown in the experimental field of the Institute of Cotton Research, Chinese Academic Agricultural Sciences (ICR, CAAS, Anyang, Henan Province, or Sanya, Hainan Province).

### Creation of *CU* mutant and construction of F_2_ population

In order to obtain abundant mutants with novel traits, approximately 15,000 seeds of Shixiya 1 were presoaked in phosphate buffer (100 mM, pH 7.0) for 12 h at 28°C. Then, the seeds were soaked in EMS (0.6%, ethyl methanesulfonate) phosphate buffer for 8 h in the dark at 28°C with continuous flipping and shaking during the period. Subsequently, the seeds were rinsed three times with distilled water for 30 min to remove residual EMS. Finally, they were sown in the experimental field of ICR, CAAS (Sanya). The M_2_ seeds were harvested by collecting one boll per plant from the surviving 7987 M_1_ plants (53.3% survival). After four generations of self-fertilization, 123 individual lines with visible phenotypes were identified out of 5,980 M_7_ plants, and one of which exhibited curly leaf and was named as *CU*.

The *CU* and DQJ were crossed to construct an F_2_ population (60 F_1_ lines) in 2020, and an F_2_ population (1,100 plants) was generated by self-fertilization of the F_1_ population in 2021.

### Super bulked segregant analysis sequencing

Fresh young leaves were collected from parental lines (DQJ and *CU*), 50 F_2_ individual plants exhibiting curly leaf and 50 F_2_ individual plants exhibiting flat leaf. Total DNA was extracted from the leaves following the previously reported method (Ali et al., 2019). Two DNA pools were constructed. One pool consisted of DNAs from 50 F_2_ plants showing the dominant phenotype (curly leaf), and another pool consisted of DNAs from 50 F_2_ plants showing the recessive phenotype (flat leaf). The experimental process is performed according to the standard protocol provided by Illumina for bulk segregation analysis (BSA)-sequencing. The DNA of each sample was randomly broken into 350-bp fragments by ultrasonic fragmentation to construct the sequencing library. The library was sequenced by Illumina HiSeq after passing the quality inspection. Each sample was sequenced at 30× coverage of the assembled genome with 150-bp paired-end reads. Clean reads were mapped to the Shixiya 1 genome ( *https://www.cottongen.org/species/Gossypium_arboreum/CRI-A2_genome_v1.0* ) by BWA after filtering the raw reads. According to the positioning results of clean reads in the reference genome, Picard was used to mark duplicates and GATK for local realignment, base recalibration, and other preprocessing to ensure the accuracy of the detected SNPs. The single-nucleotide polymorphism was further detected by GATK and filtered to obtain the final SNP site set. BSA-sequencing analysis was performed using the runQTLseqAnalysis method of QTLseqr for SNP ratio calculation. A 1,000-kb window spans multiple gene-regulatory units—an optimal scale for quantitative trait locus (QTL) scans—and the 10-kb step size yields 99% inter-window overlap to ensure continuous signal capture. Accordingly, we calculated the average ΔSNP index based on a 1,000-kb sliding window with a 10-kb step size to identify regions associated with the target trait.

### Design and detection of polymorphic molecular markers

InDel markers are specific PCR primers designed based on nucleotide insertions or deletions (InDels) identified between parental genomes. First, the genomic positions of InDels are extracted from the Variant Call Format (VCF) file generated through BSA-seq using a Perl script. Subsequently, a local Perl script retrieves the flanking sequences surrounding each InDel locus. Finally, primers are designed using Primer 3.0 software. For InDel marker polymorphism screening, PCR products were generated using the 3G Taq Master Mix for PAGE (Red Dye) kit and subsequently resolved through 8% non-denaturing polyacrylamide gel electrophoresis.

### Development and utilization of KASP maker in the *GaIAA14* cloning

Based on the BSA-seq results, a significant single signal was identified on Chr03, indicating that the curly leaf trait is controlled by a single locus. Combined with the Indel marker mapping results, KASP (Kompetitive Allele Specific PCR) markers were designed within the candidate region (Chr03: 4.31 Mb to 4.52 Mb) by HuaZhi Biotechnology Co., LTD. The development of KASP markers followed the workflow established in previous studies (Liu et al., 2023).

### RNA-seq and RT−qPCR analysis

The leaves in the different stages T1 (the first true leaf at the pre-curling stage), T2 (the first true leaf in the process of curling) and T3 (after the curling degree of the first true leaf stabilized) were taken from the wild type Shixiya 1 and *CU* mutant, respectively. Three biological repeats were used for total RNA extraction with Tiangen RNAprep Pure Plant Plus Kit (DP441). Two cDNA libraries were constructed following Illumina standard protocol and sequenced on a HiSeq 2000 platform. All paired-end reads were aligned to the *G. arboreum* reference genome through the Bowtie software (Li et al., 2009; Langmead and Salzberg, 2012). Differentially expressed genes (DEGs) were analyzed by Cufflinks software for FPKM (fragments per kilo bases per million reads) calculation (Trapnell et al., 2010).The differentially expressed genes (DEGs) between Shixiya 1 and *CU* mutant were identified using the criteria of |log2^Fold Change^| ≥ 1 and P-value ≤0.05. The KAAS (https://www.genome.jp/tools/kaas/) was used for pathway annotation analysis of the DEGs. The RT-qPCR assays were performed by the TB Green^®^ Fast qPCR Mix (Takara, RR430B). The sequences of each pair of primers used for the different RT-qPCR assays are listed in the **Supplementary Table S1**, and *His3* was used as the endogenous reference gene for the relative quantitation of the gene expression data (Gong et al., 2017). The RT-qPCR analysis was conducted with three biological replicates, followed by the application of the 2^−ΔΔCt^ method to quantify the relative gene expression level (Pfaffl, 2001; Livak and Schmittgen, 2001).

### Microscopy observation of leaf in Shixiya 1 and *CU*

To observe the differences in cellular structure within the leaves between Shixiya 1 and *CU*, the third to the last leaf was taken from the wild-type Shixiya 1 and the mutant line *CU*, respectively. The samples were fixed in 4% (w/v) paraformaldehyde, 0.25% (w/v) glutaraldehyde, and 50 mM sodium phosphate (pH 7.2), dehydrated by passage first through an ethanol series and then through a xylene ethanol series, embedded in paraffin, and finally sectioned to 20 μm by using a Leica RM2235 rotary microtome. The paraffin sections were stained with safranin stain solution and observed under an Olympus BX53 microscope.

### Virus-induced gene silencing assay

For the virus-induced gene silencing (VIGS) assay, we used the TRV vector (i.e., pTRV1 and pTRV2), along with VIGS positive control pTRV2–*PDS* and negative control (NC) empty vector pTRV2. A 294 bp *GaIAA14* fragment was cloned from the leaf of Shixiya 1 cDNA with the pTRV2-*GaIAA14* F/pTRV2-*GaIAA14* R primer pair (**Supplementary Table S1**). The pTRV1 helper plasmid, pTRV2, pTRV2-*PDS*, and pTRV2-*GaIAA14* were all introduced into the *Agrobacterium* strain GV3101. VIGS assay and silencing efficiency check were performed with *CU* mutant as donor plant according to the previously published protocol (Gong et al., 2017).

## Results

### Identification of the curly leaf mutant

**Figure 1:**
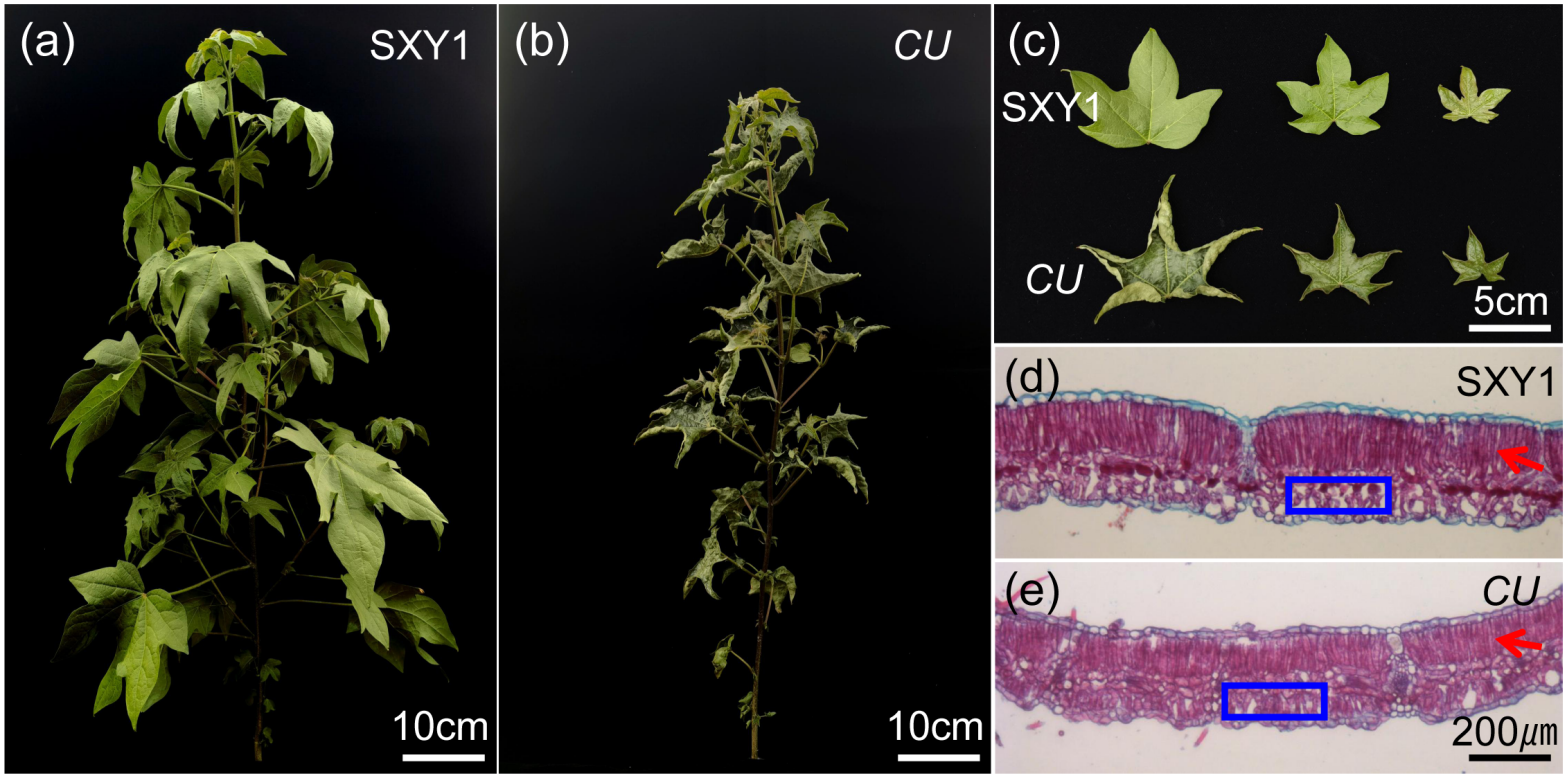
Phenotype characterization of the *CU* mutant. **(a, b)** Plant architecture of the wild-type Shixiya 1 and the *CU* mutant (scale bar = 10 cm). **(c)** Plant leaf morphology of the wild-type Shixiya 1 and the *CU* mutant (scale bar = 5 cm); **(d, e)** Longisection analysis of the third to the last leaf in the wild-type Shixiya 1 and the *CU* mutant. The red arrows mark the palisade tissue, and the blue boxes mark the spongy tissue (scale bar = 200 µm).

The curly leaf mutant was obtained from wild-type Shixiya 1 by EMS mutagenesis, and its trait was stably inherited after multiple generations of self-purification. Here, it was designated as the *CU* (curly leaf) mutant. Compared with the wild-type Shixiya 1, the plant height of the *CU* mutant was slightly reduced (**Figures 1a, b**). Notably, the leaves of the *CU* mutant were cup-curled, and the leaves’ surface was bumpy and darker (**Figure 1c**). Furthermore, we sliced the third to the last leaf of *CU* mutant and Shixiya 1 to analyze the cellular structure difference under the microscope during development. Compared with Shixiya 1, the *CU* mutant exhibited more developed and tightly packed spongy tissue, along with thinner palisade tissue (**Figures 1d, e**). The abnormal development of spongy tissue may be one of the reasons for the formation of cup-curled and bumpy leaves in the *CU* mutant.

### The *CU* locus is located on chromosome A03

**Figure 2:**
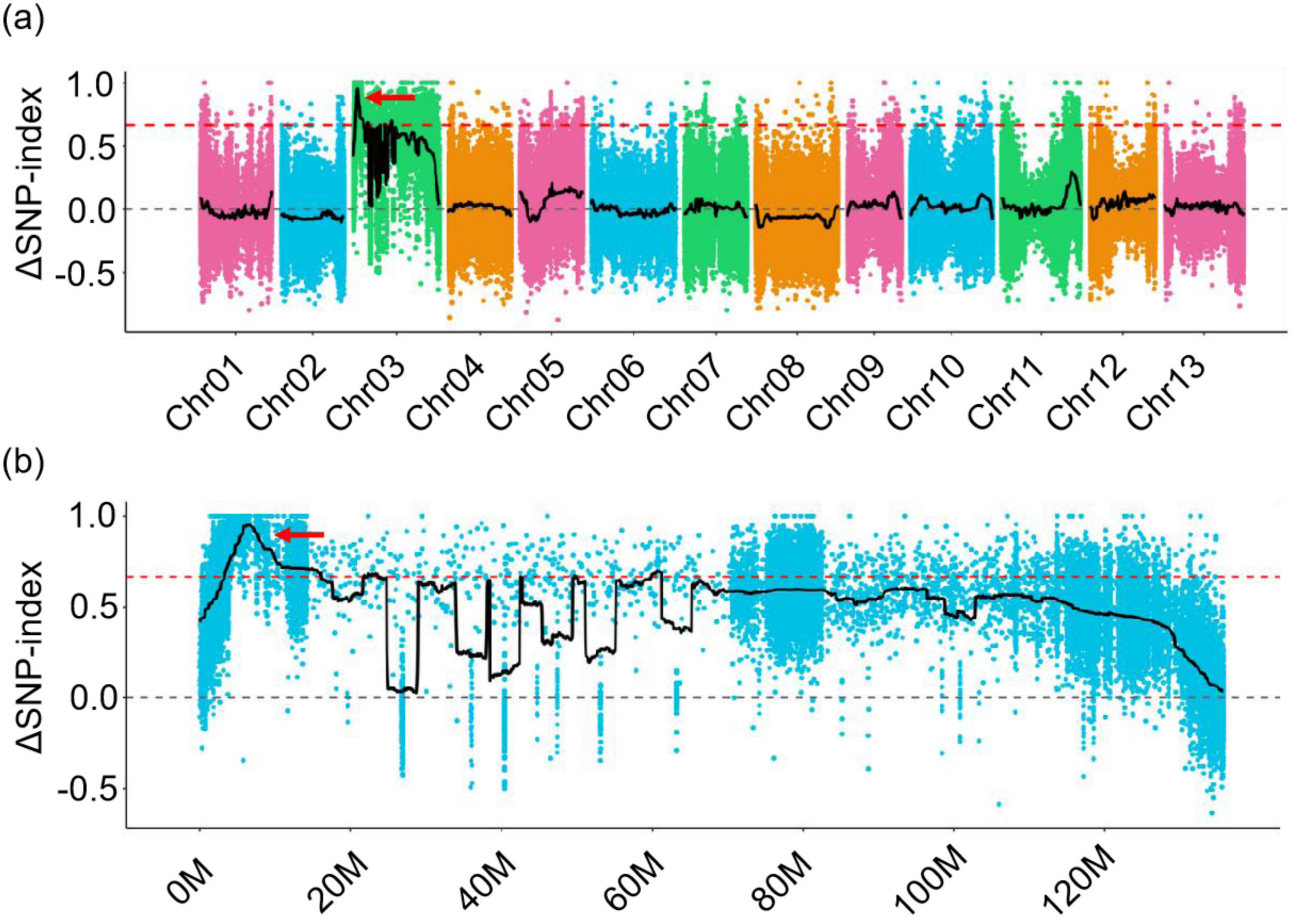
The *CU* locus is located on chromosome A03. **(a)** The *CU* locus was mapped on chromosome A03 by QTL-Seq analysis. The red arrow indicates the only window with a ΔSNP value exceeding the 95% significance threshold confidence interval across the whole genome. **(b)** Enlarged view of chromosome A03. The red arrow indicates the candidate region on the chromosome.

To identify the causal locus for the curly leaf of *CU* mutant, a *G. arboreum* line DQJ (flat leaf) and the *CU* (curly leaf) were used as parents to construct a F2 population through hybridization and selfing. All F_1_ plants exhibited curly leaf, but less curly than those of *CU* mutants (**Supplementary Figure S1**), indicating that the curly leaf allele is semi-dominant. The F_2_ population produced a 1:2:1 segregation ratio of curly leaf, moderately curly leaf, and flat leaf phenotypes (**Supplementary Table S2**), indicating that a single locus controlled the curly leaf of *CU* mutant. Furthermore, we identified the causal locus by bulk segregation analysis (BSA), and the results showed that the ΔSNP index greater than 95% confidence intervals was located on chromosome A03 from 3.30 to 16.10 Mb (**Figures 2a,b**) by aligning the sequence reads to the reference Shixiya 1 genome ( *https://www.cottongen.org/species/Gossypium_arboreum/CRI-A2_genome_v1.0* ), demonstrating that the locus controlling the curly leaf is located on chromosome A03.

### Genetic mapping of the *CU* locus to a 48.2-kb genomic region

**Figure 3:**
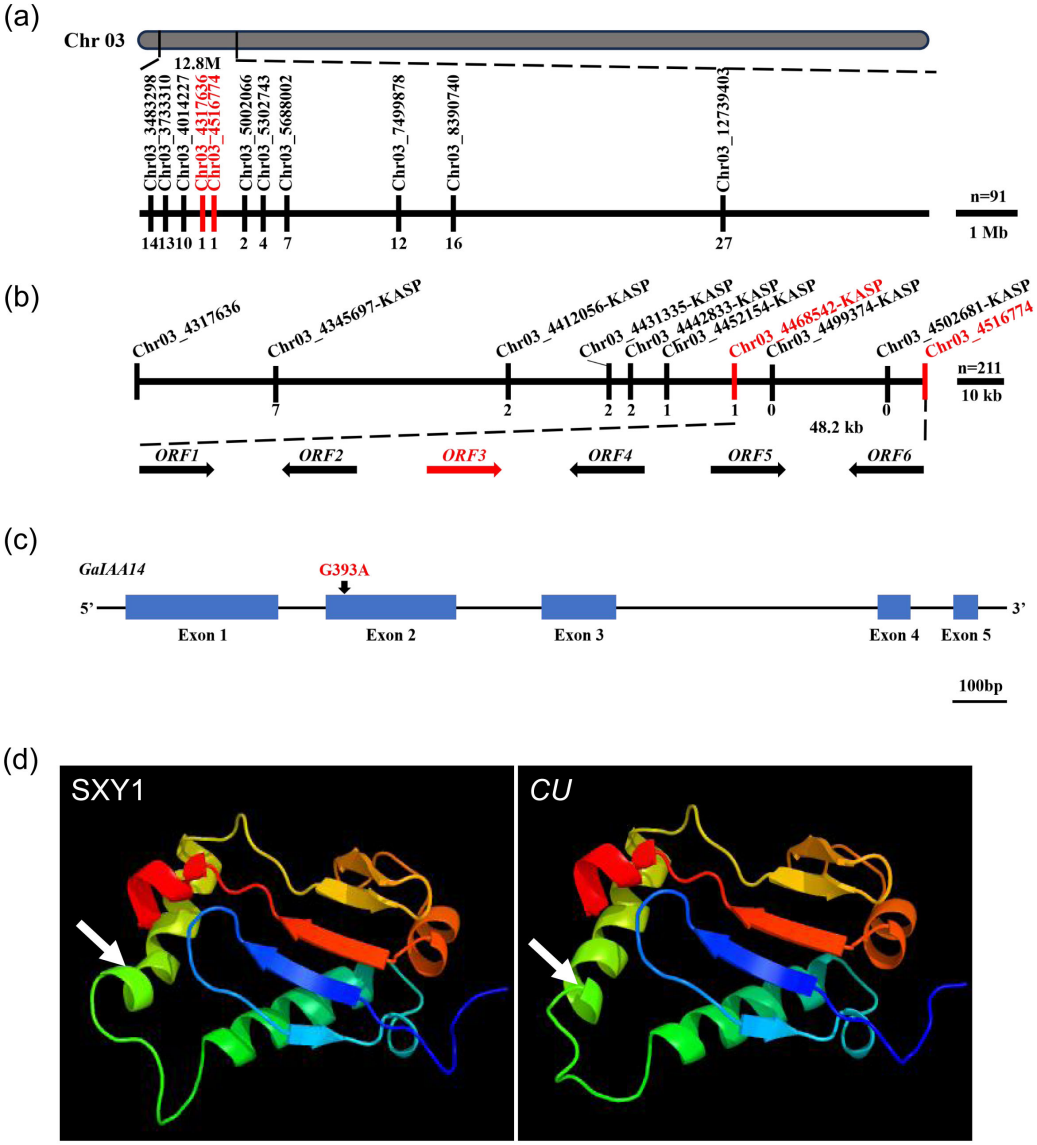
Fine mapping and cloning of *CU*. **(a)** Fine mapping of the *CU* locus in a population with 91 BML × *CU* F_2_ dominant individual plants by InDel primers, and the number of recombinants is shown below the black line. **(b)** Genetic mapping of the *CU* locus in a population with 211 DQJ × *CU* F_2_ recessive individual plants by KASP, and six ORFs located in the 48.2-kb candidate region are indicated by arrows. The number of recombinants is shown below the black line. **(c)** Single-nucleotide mutation from G to A in the second exon of GaIAA14 in the *CU* mutant. **(d)** 3D protein structure of GaIAA14 in the wild-type Shixiya 1 and the *CU* mutant. The white arrows indicate the absence of a segment of the α-helix in the *CU* mutant.

We further designed the molecular markers in the 12.8-Mb region on the basis of InDels between the two BML (BaiMiLa) and *CU* parents. A total of 70 markers were designed, and 24 markers showed significant polymorphisms with PCR between the two parents that were used for fine mapping in a BML × *CU* F_2_ population (**Supplementary Table S3**). Finally, the curly leaf locus (*CU*) was narrowed down to a 199.10-kb region flanked by Chr03_4317636 and Chr03_4516774 (**Figure 3a**). Based on the above-cited candidate interval, we first developed 13 KASP markers covering the genomic region from Chr03_4345697-KASP to Chr03_5016706-KASP (**Supplementary Table S4**). Using these markers to perform genotype testing in 211 recessive lines in the DQJ × *CU* F_2_ generation population, we found that the curly leaf trait was closely linked to the Chr03_4468542-KASP~Chr03_4516774 interval located at the 48.2-kb genomic region (**Figure 3b**).

### Cloning of the *GaCU*

There are six genes in the region from 4468542 to 4516774 on chromosome A03 of *G. arboreum* (**Supplementary Table S5**) (*Cotton Functional Genomics Database:* *https://cottonfgd.org/* ). We aligned the sequencing data of the six candidate genes between the two parents. The results showed that *Ga03G0409* had an SNP in the second exon (**Supplementary Figure S2c**), *Ga03G0407*, *Ga03G0410*, and *Ga03G0411* had SNPs at the 2000 bp region upstream of the start codon (**Supplementary Figures S2a, d, e**), and there was no difference in the nucleotide sequence of other genes between the two parents (**Supplementary Figures S2b, e**). Furthermore, the RNA-Seq analysis indicated that *Ga03G0407*, *Ga03G0410*, and *Ga03G0411* did not show a clear alteration in transcription between Shixiya 1 and *CU* mutant. Therefore, we identified *Ga03G0409* as the potential candidate gene of *CU* and then cloned and sequenced the putative ORF from RNA. Compared with Shixiya 1, a single-nucleotide mutation from G to A at the 301st position of the second exon occurred in the *CU* mutant (**Figure 3c**; **Supplementary Figure S3a**), which resulted in a valine to isoleucine (V101I) mutation in the Ga03G0409 protein (**Supplementary Figure S3b**). The gene was annotated as *IAA14* after the sequence alignment analysis, in which a single amino acid substitution occurs at the conserved degron motif GWPPV(I) within its domain II, resulting in the change from GWPPV to GWPPI. Further structural analysis of the protein revealed that the IAA14 protein in the *CU* mutant lacked a segment of α-helical structure (**Figure 3d**). This structural alteration likely impaired the normal function of IAA14, ultimately leading to leaf curling in cotton plants.

### Silencing of *GaIAA14* led to flat leaves in the *CU* mutants

**Figure 4:**
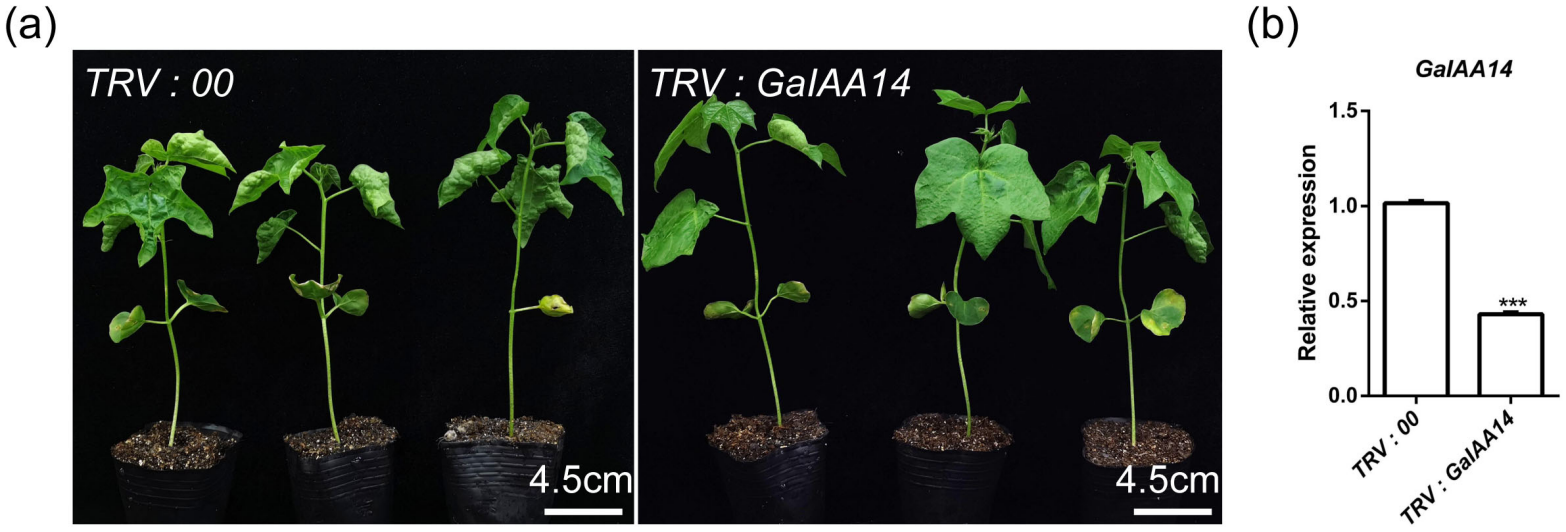
Silencing of *GaIAA14* in *CU* mutant results in leaf flattening. **(a, b)** The level of *GaIAA14* transcript in the leaves of *GaIAA14*-silenced (TRV: *GaIAA14*) plants and the negative control (TRV: *00*). Two-tailed Student’s *t*-test was used for paired comparison of the *GaIAA14* gene in TRV: *GaIAA14* and TRV: *00* leaves (***P < 0.001 or **P < 0.01 or *P < 0.05).

To further test the function of GaIAA14, a 294-bp fragment of *GaIAA14* was cloned and inserted into pTRV2 for VIGS. At 2 weeks post-infiltration of the *CU* mutants, the TRV: *GaIAA14* displayed flat leaves compared to that maintained curly leaves with a blank TRV: *00* vector (**Figure 4a**). The RT-qPCR analysis revealed that the expression of *GaIAA14* was much lower in *GaIAA14*-silenced plants compared with that in blank vector control TRV: *00* plants (**Figure 4b**), indicating that *GaIAA14* is the key gene for the curly leaf phenotype associated with the *CU* locus.

### Analysis of differentially expressed genes between *CU* and Shixiya 1

**Figure 5:**
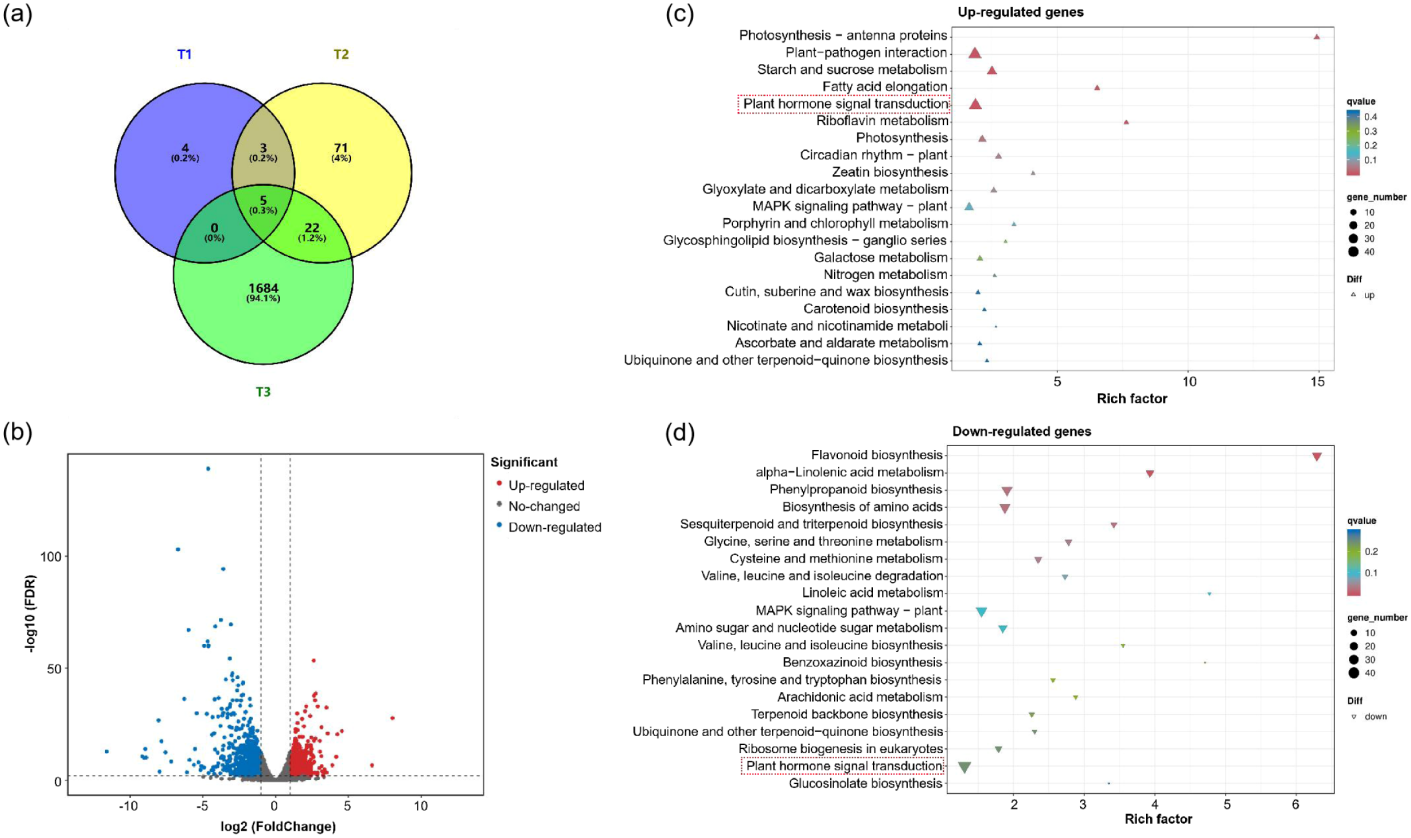
Transcriptomic comparison of the *CU* mutant versus Shixiya 1. **(a)** Venn diagram showing the overlaps between the different stages of the *CU* mutant and Shixiya 1. The number above each stage designation is the total differentially expressed genes (DEGs) detected in that stage(s). **(b)** Volcano plot of differentially expressed genes between the *CU* mutant and Shixiya 1 at T_3_ stage (after the curling degree of the first true leaf stabilized). **(c)** Top 20 KEGG enriched pathways of upregulated genes. **(d)** Top 20 KEGG enriched pathways of downregulated genes. The top 20 KEGG enriched pathways are selected by p. adjust value sorting. Count, the bubble size, represents the number of enriched genes. Rich factor represents the multiple of enrichment level of differentially expressed genes in a target pathway relative to the entire genomic background.

*GaIAA14*, a member of the AUX/IAA gene family, is an early auxin response factor (Zhao et al, 2021). Theoretically, the mutations of *GaIAA14* would affect the auxin signaling pathway. Therefore, RNA-Seq was performed with the first true leaf of Shixiya 1 and *CU* at different stages: T1 (the first true leaf at the pre-curling stage), T2 (the first true leaf in the process of curling), and T3 (after the curling degree of the first true leaf stabilized). In total, 1,789 gene transcripts were identified. Notably, the stage-specific DEGs (1,684) in the T_3_ stage were significantly more than that in the T_1_ (4) and T_2_ (71) stages (**Figure 5a**), indicating that the stage-specific genes involved in regulating leaf curling in the *CU* mutant were predominantly active during the T3 stage. A total of 1,711 differentially expressed genes (DEGs) were identified in the T_3_ stage, including 760 upregulated genes and 951 downregulated genes (**Figure 5b**; **Supplementary Table S6**). To further understand the molecular functions of these DEGs in the process of cotton leaf morphogenesis, the KEGG enrichment analysis was conducted at the T_3_ stage. The results showed that both upregulated and downregulated genes were associated with plant hormone signal transduction pathways, including 40 and 42 DEGs, respectively (**Figures 5c, d**; **Supplementary Table S7**). In plant hormone signal transduction pathways, the auxin signaling pathway contained the highest number of genes, totaling 15 genes (six upregulated DEGs and nine downregulated DEGs). These included six AUX/IAA family members, four auxin response factors (ARFs), three GH3 auxin-responsive promoters, and two auxin transporter-like proteins (**Supplementary Table S7**). Collectively, these results imply that *GaIAA14* regulates cotton leaf morphology, which is potentially dependent on auxin distribution and signaling.

## Discussion

Leaf morphology is important for cotton growth and yield. The curly leaf is a significant variation in leaf morphology (Wu et al., 2025). Moderate leaf curling helps improve plant canopy structure, reduce transpiration, and thereby enhance effective light energy utilization and stress resistance (Li et al., 2017; Wu et al., 2010). Previous studies have demonstrated that the curled leaf mutants found in *Gossypium arboreum* ‘Xiaobaihua’ (Asian cotton) and *Gossypium hirsutum* ‘Stoneville 2B’ (upland cotton) are respectively controlled by a pair of recessive genes, manifesting as upward and inward curling of the leaf margins (Yu, 1939; Di et al., 2016). Here we obtained a heritable curly leaf mutant (*CU*) through EMS mutagenesis. The genetic analysis revealed that the *CU* mutant represents a gain-of-function mutation, and its curly leaf phenotype is controlled by a single gene as a semi-dominant trait. Within the *CU* locus, the *GaIAA14* gene plays a key role in regulating curly leaf of cotton. Silencing *GaIAA14* in the *CU* mutant restored cotton leaves to a flat phenotype. A single-nucleotide mutation from G to A at the 301st positions in *GaIAA14* resulted in an amino acid substitution from valine (V) to isoleucine (I). The substitution at amino acid position 101, located in the domain-II Degron motif (GWPPV) and conserved in most plants, is key to the phenotypic mutation (Jain and Khurana, 2009). Amino acid variations in this region often result in dominant or semi-dominant mutations, producing gain-of-function mutants (Tan et al., 2023; Huang et al., 2020; Wei et al., 2021; Cheng et al., 2019). The prediction and analysis of the 3D structure of GaIAA14 revealed that the IAA14 protein in the *CU* mutant lacks a segment of an alpha-helix, likely impairing its function. Existing studies indicate that specific mutations of any amino acid within the “GWPPV(I)” motif can potentially lead to the accumulation of IAA proteins, with the V > A mutation known to cause IAA protein accumulation (Ramos et al., 2001). However, no reports documented IAA protein accumulation resulting specifically from a V > I mutation. Based on this, we speculate that the V > I mutation would also lead to the accumulation of the IAA14 protein, thereby producing the curly leaf phenotype.

Recent advances have been achieved in understanding the molecular mechanisms of leaf curling in plants. Multiple genes (e.g., *YABBY*, *KANADI*) and phytohormones (e.g., auxin, abscisic acid [ABA]) play critical roles in regulating leaf curling (Zhao, 2010; Jiang et al., 2025). Auxin-responsive genes (e.g., ARF, AUX/IAA, SAUR, and GH3) directly regulate leaf morphogenesis by modulating asymmetric cell division and elongation across epidermal layers (Lee et al., 2024; Hussain et al., 2021). In this study, we performed an RNA-Seq with the leaves in *CU* mutant and Shixiya 1. The result of the KEGG enrichment analysis showed a significant differential down-regulation of a large number of auxin-responsive genes and ABA-negative regulatory factors, including Aux/IAAs, ARFs, GH3s, and protein phosphatase 2Cs (PP2Cs) (**Supplementary Table S7**). This suggests that the emergence of the leaf-curling phenotype in *CU* mutants is closely related to auxin signaling pathways. The coordinated downregulation of auxin core genes (e.g., Aux/IAAs, ARFs, GH3s) in the *CU* mutant indicates a significantly reduced efficiency of auxin signal transduction. This leads to obstruction of the auxin response pathway, impairing polar cell elongation in leaves and vascular development. Concurrently, the downregulation of PP2Cs may enhance ABA signaling sensitivity, promoting stomatal closure and cellular contraction. These effects synergize with auxin deficiency to exacerbate leaf curling. Combined with the analysis of the cell structure of leaf tissues, we propose that abnormal auxin distribution drives the excessive expansion of spongy tissue in the *CU* mutant leaves, with reduced intercellular spaces, restricting cell elongation but increasing turgor pressure, causing the faster expansion of the abaxial side than the adaxial side and resulting in upward leaf curling. Additionally, the thinned palisade tissue weakens the mechanical support on the adaxial side, further exacerbating curl formation due to spongy tissue expansion.

The key role and editing site of *IAA14* in cotton leaf curly were identified for the first time, which confirmed the conservative role of *IAA* gene and the auxin pathway in leaf morphological regulation. It also provides a theoretical basis for improving cotton traits and adaptability by accurately regulating the IAA signaling pathway through gene editing technology (such as CRISPR-Cas9). Upcoming research will further strengthen investigations into the interplay between IAA and other hormones (e.g., abscisic acid, cytokinins) during leaf morphogenesis, delve deeper into the linkages between leaf curling mechanisms and crop yield/quality traits, and provide theoretical foundations for sustainable agricultural development.

## Data Availability

The original contributions presented in the study are included in the article/**Supplementary Material**, and all the data sets generated during the current study are available in the NGDC BIG Submission Portal under project number PRJCA042233 for RNA-seq. Further inquiries can be directed to the corresponding authors.

## Supplementary material

Supplementary Figure 1 The phenotypic characteristics of F_1_ progeny from the DQJ × *CU* cross (scale bar = 10 cm).

Supplementary Figure 2 Integrative Genomics Viewer (IGV) analysis for the resequencing data of the six candidate genes. The SNP sites between the parent and the reference genome were marked by red triangles.

Supplementary Figure 3 Sequence alignment of *Ga03G0409* between the *CU* mutant and wild-type Shixiya 1. **(a)** Coding sequence (CDS) alignment of *Ga03G0409* between the *CU* mutant and Shixiya 1. A single-nucleotide mutation from G to A at the 301st position was observed in the *CU* mutant, **(b)** amino acid sequence alignment of Ga03G0409 between the *CU* mutant and Shixiya 1. A valine to isoleucine (V101I) mutation in the CU protein was observed.
