# Supplementary material for: A single-nucleotide mutation of G301A in GaIAA14 confers leaf curling in Gossypium arboreum
Source: Front Plant Sci. 2025 Jul 22;16:1645239. doi: 10.3389/fpls.2025.1645239 (PMC12321824; doi:10.3389/fpls.2025.1645239)
Supplement: Supplementary file 1 [file Table1.docx]

**Table S1.** The primer in this study

| Primer | Sequence（5΄→3΄） | Application |
| --- | --- | --- |
| *Ga03G0409* QF  *Ga03G0409* QR  His3 QF  His3 QR  *Ga03G0409* F  *Ga03G0409* R  pTRV2-*GaIAA14* F  pTRV2-*GaIAA14* R | GAGACTGTTGATTTGAAGCTCAAT  ATACTTGTGCCTTGGCTGGT  TCAAGACTGATTTGCGTTTCCA  GCGCAAAGGTTGGTGTCTTC  ATGCTAGGAACTGAGCATGATTTGA  TTAGACTCTGCTCTTGCATTTCTCC  ctgtgagtaaggttaccgaattcCTGTGTCTCGGCCTGCCT  tcgagacgcgtgagctcggtaccCTGGTTAGCCATAATGTTCTTCCT | RT-qPCR  RT-qPCR  Gene clone  VIGS |

**Table S2.** Chi-square test and genetic analysis of F_2_ population

| F_2_ population | Sum | Curly-leaved plant | Moderately curly-leaved plant | Flat-leaved plant | Expected ratio | χ2 (2, 0.05) =5.99 |
| --- | --- | --- | --- | --- | --- | --- |
| DQJ × *CU* | 1072 | 271 | 537 | 264 | 1:2:1 | 0.095 |

**Table S3.** The primer sequence for InDel marker of polymorphism in the *CU* mutant

| Primer | Sequence（5΄→3΄） | Application |
| --- | --- | --- |
| Chr03_3483298F  Chr03_3483298R  Chr03_3731885F  Chr03_3731885R  Chr03_3733310F  Chr03_3733310R  Chr03_3744771F  Chr03_3744771R  Chr03_3744897F  Chr03_3744897R  Chr03_3747689F  Chr03_3747689R  Chr03_4014227F  Chr03_4014227R  Chr03_4091188F  Chr03_4091188R  Chr03_4195819F  Chr03_4195819R  Chr03_4310088F  Chr03_4310088R  Chr03_4313688F  Chr03_4313688R  Chr03_4456645F  Chr03_4456645R  Chr03_4474005F  Chr03_4474005R  Chr03_4516774F  Chr03_4516774R  Chr03_5002066F  Chr03_5002066R  Chr03_5006177F  Chr03_5006177R  Chr03_5055823F  Chr03_5055823R  Chr03_5090876F  Chr03_5090876R  Chr03_5208027F  Chr03_5208027R  Chr03_5258804F  Chr03_5258804R  Chr03_5302743F  Chr03_5302743R  Chr03_5315259F  Chr03_5315259R  Chr03_5317856F  Chr03_5317856R  Chr03_5433525F  Chr03_5433525R | AAGTAGAGGGGCCAATGTTTTA  GACAATCTAAGCACCGGTTCAA  CAATCATGGCCATTTTGACAAA  TCTATAACAGGGCGGAGTAAAG  AAGAACAACCCCGTAGCTATAG  TGCCCCAATCTAAGTCCATGTC  CAACAGCCTTCTCCGCCATCAA  CCATTTTCTAAACGGGCCTCAT  GCACATCAGTTAGCCACCATTC  TTAATATTAAAATCGGGCCGGG  CGAAAATGGGCGATGGTCAAAT  GGTTGATGAGGCTAATGTTGAT  AGGTGGACGGAGCTGGACT  TCATGGGTTAAGTTGGGCCTTA  GTTGTCAACTACCTTTGGATCC  TCGGTCGAAACTAAACCTAATT  TTCCACGCACAGTAATCTAGAA  GGCTACGAACGAGTCTCAAGAA  TGGCAAGAAAACATGGGATTGA  ACCACCTTCGATCCAAACTAAC  TGGCAAAACAGTAAACAAACAA  CGGTTCGAAGGCTTATCTGAAA  AATCACTAGCAAAAGCAAGACT  ACCACCCACAGTTTCTTTTAGA  TGTCCCTGTCTAACCTGTGAAA  CCGCCCAGCCCATGATCAG  TGCGGCTTAGGAATGGGTATAA  TCAGCAAAAGTTCCAATGACAA  TGATAGGACGGCAACACACAAA  ATGTCCAGTGGCACCAGTATGT  GCCCTTTACATATGCTCAAGTT  GATGTAGTTGCGAAGGGTTAGA  TCATCCAGAACAGTCGGCTTAA  GAGCTGCGGTCTGAAACTTTTA  GGGCGACGCACCTAATGTTTCT  TTCCCTCGGCCTCAAAGTACTG  CACTGAGAGGTACGGGGATATA  TCGGCTTGGGTTTCTTCATATA  TGGTGATTCGGCCGCATGAAGA  TGTGAATGGGTGCGCAGATCCT  TGCTGCCATTTGTGTTGCTAAA  ACACACACACACACACACAGTT  GCTCAGTTGTCGGGAATGGTTA  GCCACCAGTGTATGAGCCTAAG  TGCATTGACAGTTTGGAAGTTT  ACTCTCAATCATGTCTCAACTC  TCCTATCGACATTGATGGATTG  CGACCCAATCTGAGTCACATTA | InDel marker  InDel marker  InDel marker  InDel marker  InDel marker  InDel marker  InDel marker  InDel marker  InDel marker  InDel marker  InDel marker  InDel marker  InDel marker  InDel marker  InDel marker  InDel marker  InDel marker  InDel marker  InDel marker  InDel marker  InDel marker  InDel marker  InDel marker  InDel marker |

**Table S4.** The primer sequence for KASP marker of polymorphism in the *CU* mutant

| Primer | Sequence（5΄→3΄） | Application |
| --- | --- | --- |
| Chr03_4345697F_FAM  Chr03_4345697F_HEX  Chr03_4345697R  Chr03_4412056F_FAM  Chr03_4412056F_HEX  Chr03_4412056R  Chr03_4431335F_FAM  Chr03_4431335F_HEX  Chr03_4431335R  Chr03_4442833F  Chr03_4442833R_FAM  Chr03_4442833R_HEX  Chr03_4452154F  Chr03_4452154R_FAM  Chr03_4452154R_HEX  Chr03_4468542F_FAM  Chr03_4468542F_HEX  Chr03_4468542R  Chr03_4499374F  Chr03_4499374R_FAM  Chr03_4499374R_HEX  Chr03_4502681F_FAM  Chr03_4502681F_HEX  Chr03_4502681R  Chr03_4772027F_FAM  Chr03_4772027F_HEX  Chr03_4772027R  Chr03_4839552F_FAM  Chr03_4839552F_HEX  Chr03_4839552R  Chr03_4899303F  Chr03_4899303R_FAM  Chr03_4899303R_HEX  Chr03_4982340F  Chr03_4982340R_FAM  Chr03_4982340R_HEX  Chr03_5016706F  Chr03_5016706R_FAM  Chr03_5016706R_HEX | GAAGGTGACCAAGTTCATGCTAACTTTCGAATAGCTCGGAAACTATTTTG  GAAGGTCGGAGTCAACGGATTAACTTTCGAATAGCTCGGAAACTATTTTA  AAGCTTTGATCACTTGACTTCAAAAGG  GAAGGTGACCAAGTTCATGCTGAGATTTGAAATGGGGATGTGAATTAT  GAAGGTCGGAGTCAACGGATTAGATTTGAAATGGGGATGTGAATTAA  TCTTCCCGCCCTATTATAAAATTCTC  GAAGGTGACCAAGTTCATGCTATCCTGGTAAATGCAGAAATGAACAC  GAAGGTCGGAGTCAACGGATTCCTGGTAAATGCAGAAATGAACAA  TAGAGGTTTCTCTTGAGGTTCAGCAT  AGGAGCAATTCCCAATAGATCTTTTC  GAAGGTGACCAAGTTCATGCTATCCTTGAAAATTAGAACTCACACGTG  GAAGGTCGGAGTCAACGGATTAATCCTTGAAAATTAGAACTCACACGTT  GCACCCATCGTTTTAAACTAAAGCTA  GAAGGTGACCAAGTTCATGCTGACAGCAATGTTTCAACCATGAG  GAAGGTCGGAGTCAACGGATTGACAGCAATGTTTCAACCATGAA  GAAGGTGACCAAGTTCATGCTGTTCGACTGGAAAAAAAAGTTCTACGTA  GAAGGTCGGAGTCAACGGATTCGACTGGAAAAAAAAGTTCTACGTG  TGGTGTGACGAACATATATAGGACGT  GTTGGATTTTAAGGGCACAAGTATTG  GAAGGTGACCAAGTTCATGCTGCCATAATGTTCTTCCTATAAGATCGAAC  GAAGGTCGGAGTCAACGGATTGCCATAATGTTCTTCCTATAAGATCGAAT  GAAGGTGACCAAGTTCATGCTTCGATAAGTTGAGGTGATAGTTAACTCGA  GAAGGTCGGAGTCAACGGATTCGATAAGTTGAGGTGATAGTTAACTCGG  AACCCTCCCAATAAGTAATTTTCTCG  GAAGGTGACCAAGTTCATGCTAGTGATCTAACCAATTCAATCACTAGTCC  GAAGGTCGGAGTCAACGGATTCAGTGATCTAACCAATTCAATCACTAGTCT  TGATGATGTGGTAATGATGTGGTACA  GAAGGTGACCAAGTTCATGCTGTTCTAGGGTTGAGAGTTGAAAAGGTAGT  GAAGGTCGGAGTCAACGGATTTCTAGGGTTGAGAGTTGAAAAGGTAGC  AACCTTCTACCACATTTACCAAAGTCC  TGTTGCCTAGTTAAGAAGGCTATCATG  GAAGGTGACCAAGTTCATGCTGGTCTTGTTGTGGTTAACCTCTTAACA  GAAGGTCGGAGTCAACGGATTGGTCTTGTTGTGGTTAACCTCTTAACG  GAAGAGTTGCCTGAACCATTTAGGTA  GAAGGTGACCAAGTTCATGCTCAATTCACCATTGCAGGGTTATTACTATA  GAAGGTCGGAGTCAACGGATTAATTCACCATTGCAGGGTTATTACTATT  ATTTTTCGAGCAAGATATTGAGGTGA  GAAGGTGACCAAGTTCATGCTTGATATCTAAACCTGCTTCCCGAC  GAAGGTCGGAGTCAACGGATTCTGATATCTAAACCTGCTTCCCGAT | KASP marker  KASP marker  KASP marker  KASP marker  KASP marker  KASP marker  KASP marker  KASP marker  KASP marker  KASP marker  KASP marker  KASP marker  KASP marker |

**Table S5.** The list of the six candidate genes

| **ORF** | **Gene ID** | **Gene Name** | **Description** | **Start** | **End** |
| --- | --- | --- | --- | --- | --- |
| 1 | *Ga03G0407* | *CIPK7* | CBL-interacting serine/threonine-protein kinase 7 | 4,470,674 | 4,472,049 |
| 2 | *Ga03G0408* | *AUX22B* | Auxin-induced protein 22B | 4,477,796 | 4,480,533 |
| 3 | *Ga03G0409* | *IAA14* | Auxin-responsive protein IAA14 | 4,498,982 | 4,500,540 |
| 4 | *Ga03G0410* | *TatB* | Sec-independent protein translocase protein TatB | 4,504,957 | 4,506,761 |
| 5 | *Ga03G0411* | *BHLH148* | Transcription factor BHLH148 | 4,512,610 | 4,513,462 |
| 6 | *Ga03G0412* | *RPS15* | 40S ribosomal protein S15 | 4,513,951 | 4,515,247 |
