# Supplementary figures and images for: A single-nucleotide mutation of G301A in GaIAA14 confers leaf curling in Gossypium arboreum
Source: Front Plant Sci. 2025 Jul 22;16:1645239. doi: 10.3389/fpls.2025.1645239 (PMC12321824; doi:10.3389/fpls.2025.1645239)

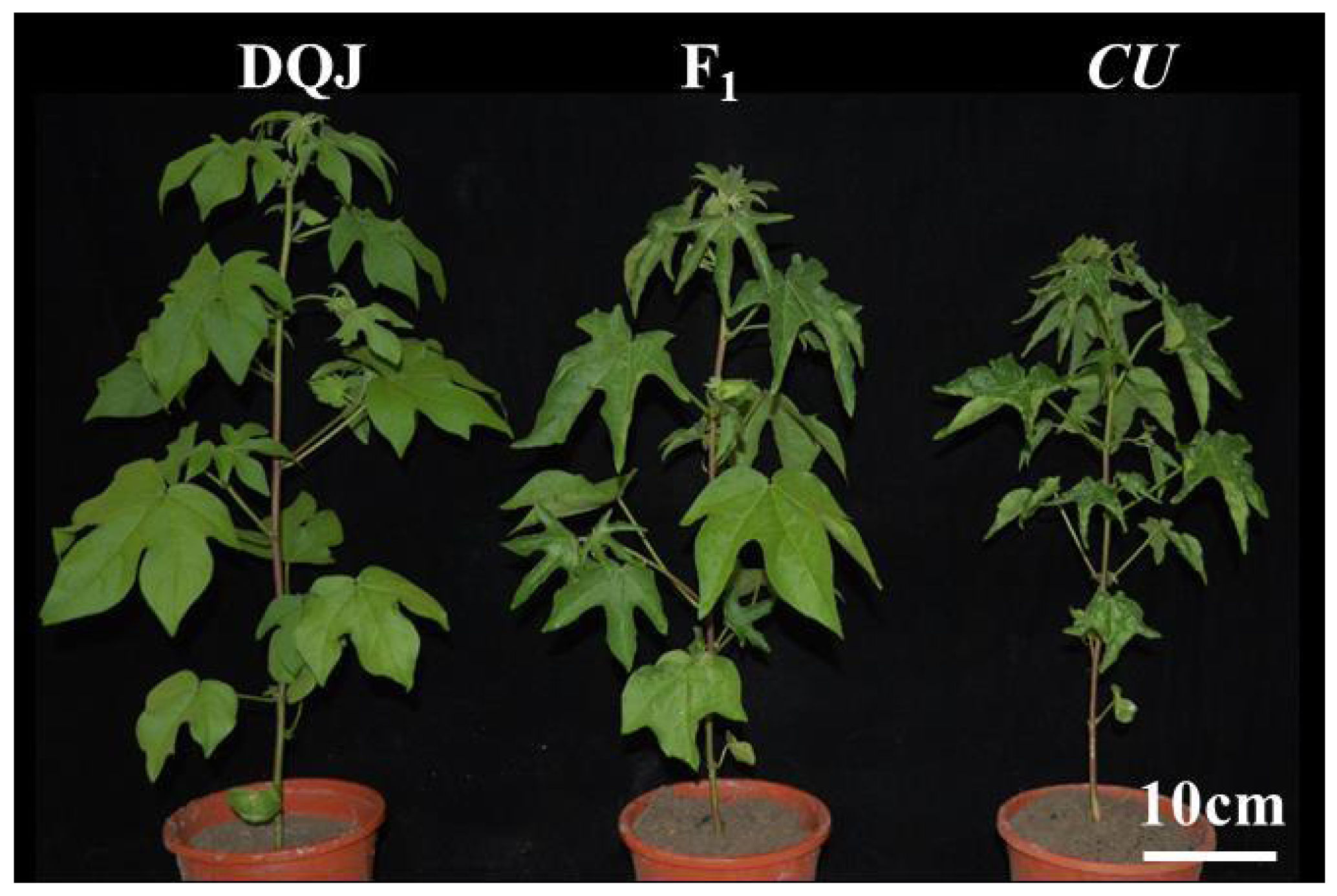

Supplement: Supplementary Figure 1 — The phenotypic characteristics of F1 progeny from the DQJ × CU cross (scale bar = 10 cm). [file Image1.tif]

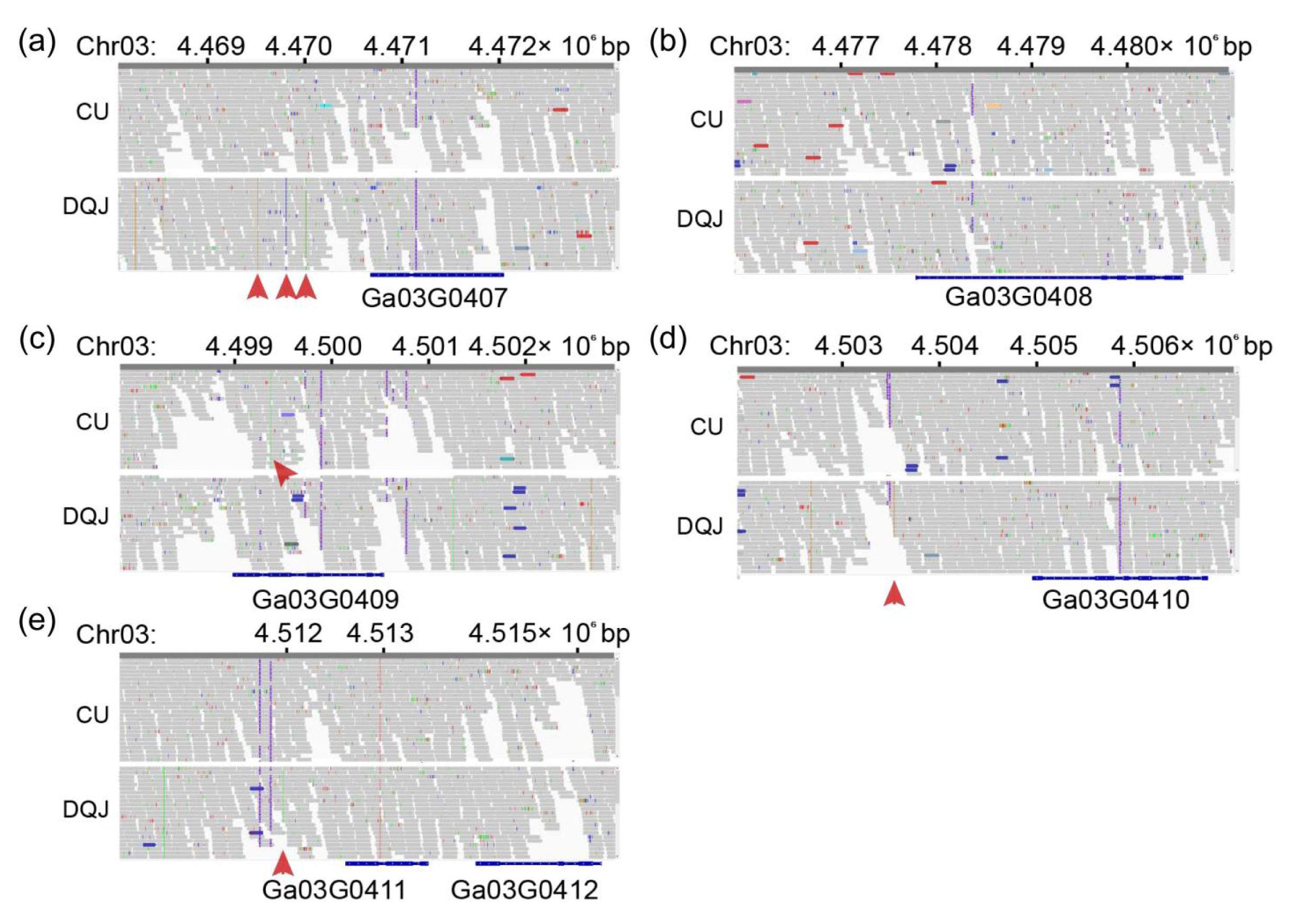

Supplement: Supplementary Figure 2 — Integrative Genomics Viewer (IGV) analysis for the resequencing data of the six candidate genes. The SNP sites between the parent and the reference genome were marked by red triangles. [file Image2.tif]

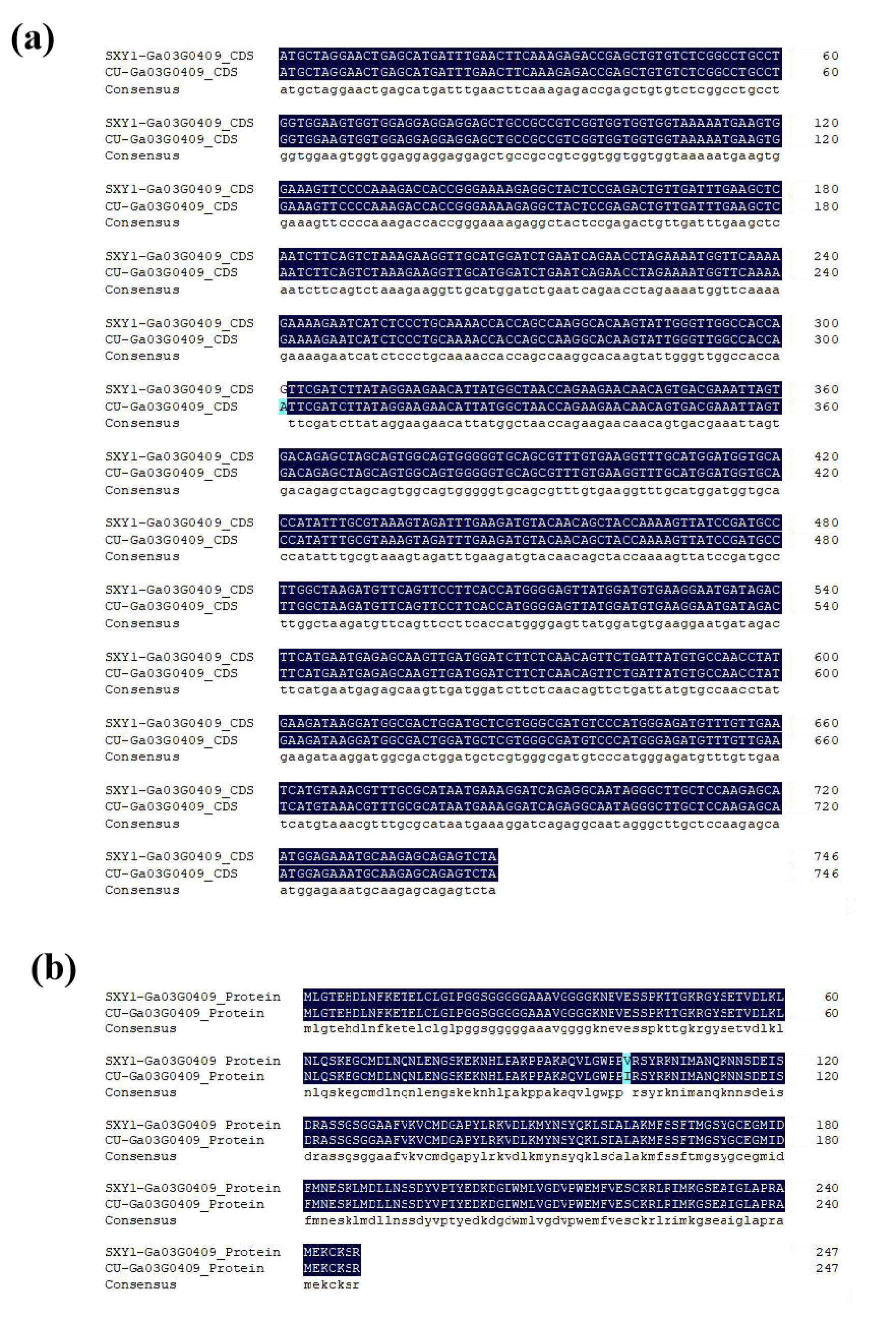

Supplement: Supplementary Figure 3 — Sequence alignment of Ga03G0409 between the CU mutant and wild-type Shixiya 1. (a) Coding sequence (CDS) alignment of Ga03G0409 between the CU mutant and Shixiya 1. A single-nucleotide mutation from G to A at the 301st position was observed in the CU mutant, (b) amino acid sequence alignment of Ga03G0409 between the CU mutant and Shixiya 1. A valine to isoleucine (V101I) mutation in the CU protein was observed. [file Image3.tif]
